# Validation of an AI-powered mobile application for personalizing medical note explanations: a mixed-methods evaluation

**DOI:** 10.3389/fdgth.2026.1771051

**Published:** 2026-08-25

**Authors:** Nicholas Lamb

**Affiliations:** Medcopywriter Ltd, Chipping Norton, United Kingdom

**Keywords:** artificial intelligence, digital health, health literacy, large language models, patient communication

## Abstract

**Introduction:**

Nearly half of adults struggle to understand written health information, making medical communication a persistent barrier to effective care. While artificial intelligence has potential to improve health communication, few patient-facing tools have undergone systematic validation for personalized medical explanation.

**Methods:**

Patiently AI is a mobile application designed to clarify clinician-authored medical notes using large language models with audience-specific adaptations (child, teenager, adult, carer) and tone variations (friendly, informative, reassuring). A three-phase mixed-methods evaluation was conducted: (1) computational readability analysis of 210 AI-generated explanations using established metrics; (2) expert review by 15 healthcare professionals assessing medical accuracy, safety, and communication quality; and (3) a patient survey of 54 participants evaluating preferences, comprehension, and acceptance.

**Results:**

AI-generated explanations demonstrated consistent improvements in readability, with mean Flesch–Kincaid Grade Level decreasing by 2.96 levels (10.57–7.61), Flesch Reading Ease increasing by 31.9 points (37.7–69.6), and Gunning Fog Index decreasing by 4.09 points (14.5–10.4); all improvements were statistically significant (all *P* ≤ 0.002). Readability gains were greatest for younger audiences (child: 4.25 grade-level reduction; adult: 1.80). Expert reviewers rated outputs highly for medical accuracy (4.49 ± 0.83/5), clarity (4.53 ± 0.77/5), and trustworthiness (4.37 ± 0.90/5), with 87.3% assessed as clinically safe. Inter-rater agreement across the 15 reviewers was substantial (Gwet's AC1 = 0.72 for safety assessments). Among patients, 70.0% of responses preferred AI-generated explanations (*P* < 0.001), with 98.1% comprehension accuracy and high ratings for clarity (4.58 ± 0.65/5) and confidence in care (4.19 ± 0.85/5). Overall, 70.4% indicated a likelihood of using the application.

**Conclusions:**

This mixed-methods evaluation suggests that a deliberately constrained, language-focused AI system can improve the accessibility of medical notes while preserving clinical accuracy and safety. Patiently AI demonstrates a scalable approach to supporting health literacy and patient engagement without extending into clinical interpretation.

## Introduction

Health literacy, defined as the capacity to obtain, process, and understand basic health information needed to make appropriate health decisions (1), remains a persistent challenge in healthcare delivery. Nearly half of adults struggle to understand written health information (2, 3), with disproportionate impacts on older adults, ethnic minorities, and individuals with lower educational attainment (4). Limited health literacy is associated with increased hospitalizations, emergency department use, medication errors, and mortality (5–7), with estimated annual costs to the United States economy of between $106 billion and $238 billion (8).

Recent policy and system-level changes have intensified this challenge. Initiatives mandating patient access to clinical records, such as the 21st Century Cures Act in the United States, reflect a broader international shift toward transparency and patient empowerment (9). As a result, patients increasingly encounter complex clinical documentation without accompanying interpretation or clarification. Medical notes, while essential for clinical care, often contain dense terminology, abbreviations, and domain-specific language that impede patient understanding and engagement (10). Traditional strategies to improve medical communication, including plain-language guidelines and clinician training, have demonstrated modest benefits but face significant scalability limitations (11, 12).

Large language models (LLMs) have introduced new opportunities to support personalized health communication at scale (13, 14). Prior work has shown promise in simplifying medical text (15, 16), translating complex information (17), and adapting communication styles (18). However, many existing evaluations have applied uniform simplification for a generic reader or focused on a single document type and outcome measure, and relatively few have evaluated AI-generated patient-facing explanations across clinical accuracy, safety, and patient acceptance simultaneously (17, 19–21).

Emerging evidence highlights both the potential and the risks of AI-generated medical communication. For example, Stanceski et al. (2024) reported improved readability of AI-generated discharge instructions but identified safety concerns in 18% of cases, including hallucinations and the introduction of novel medications not present in the source material (22). These findings underscore the risks associated with unconstrained or interpretive AI systems in clinical contexts. Documented and potential risks of AI-mediated patient communication extend beyond hallucination and factual error to include oversimplification that underplays clinical seriousness, omission of important context, propagation of errors at scale, depersonalization of care with reduced human interaction and empathy, privacy violations where identifiable information is processed by third-party systems, and biased or unfair communication across demographic groups (15, 16, 23). Effective medical communication requires more than text simplification alone; it demands audience-specific adaptation that accounts for age, health literacy, and role in care (patient vs. caregiver) (24, 25). Children, adolescents, adults, and caregivers differ in communication needs and information processing styles (26, 27), and emotional framing (informative, reassuring, or friendly) plays a meaningful role in engagement and comprehension (28). Most current AI applications employ uniform approaches that do not adequately address this diversity.

### Related work

AI-based simplification and summarization of clinical documentation for lay readers is a rapidly developing field. Zaretsky et al. used a LLM to transform inpatient discharge summaries into patient-friendly language, reporting marked readability gains while identifying occasional omissions and inaccuracies requiring clinician review (20). Jeblick et al. evaluated ChatGPT-simplified radiology reports, which radiologists judged largely factually correct and complete, though with instances of incorrect or potentially harmful text (21). Koohi Habibi Dehkordi et al. developed an effective sequential prompting approach for simplifying electronic health records with LLMs for patients without medical backgrounds (29), and subsequently showed that adding concept highlights to discharge notes improved the completeness and correctness of GPT-4o-generated summaries (30). Stanceski et al. evaluated generative AI discharge instructions, reporting improved readability alongside safety concerns in 18% of cases (22). Collectively, this literature establishes the feasibility of LLM-based simplification of clinical documents while underscoring persistent risks of omission, error, and oversimplification. However, these studies largely apply a single simplification target rather than audience-specific personalization, rarely combine expert safety review with patient preference and comprehension evaluation, and typically evaluate research prototypes rather than deployed applications.

To address these gaps, Patiently AI was developed, a mobile application designed to clarify clinician-authored medical notes through language transformation rather than clinical interpretation. The system adapts linguistic complexity and communication tone based on user-selected parameters, generating audience-appropriate explanations for children, teenagers, adults, and carers across different emotional contexts. Each of these design constraints responds to a documented risk in the literature: restricting the system to language transformation limits hallucination and interpretive error; explicit audience and tone parameters counter uniform oversimplification and depersonalization; and the absence of stored personal health information mitigates privacy risk.

While prior studies have examined AI-assisted simplification of medical text (20–22, 29, 30), this study extends existing work in several important ways. First, it systematically evaluates audience-specific adaptations (child, teenager, adult, and carer) rather than applying uniform simplification. Second, it examines the role of emotional tone within a clinical safety framework. Third, it triangulates evidence across computational readability metrics, expert clinical review, and patient preferences. Finally, the evaluation focuses on a publicly available application rather than a research prototype, supporting practical relevance and reproducibility.

This study presents a mixed-methods validation of Patiently AI, a publicly available mobile application, using three complementary approaches: computational readability analysis, expert clinical review, and patient preference evaluation. A mixed-methods design was chosen because no single evaluation perspective is sufficient: computational metrics quantify linguistic accessibility but cannot detect clinical error; expert review assesses safety and accuracy but not lay understanding; and patient evaluation captures comprehension and acceptance but not clinical validity. Triangulating these complementary perspectives therefore provides a more comprehensive understanding of system performance than any single method alone. The objectives were to: (1) quantify readability improvements across audience adaptations; (2) assess medical accuracy, safety, and communication quality through expert review; and (3) evaluate patient preferences, comprehension, and acceptance of AI-generated explanations.

## Methods

### Study design

A prospective, mixed-methods evaluation was conducted to assess the Patiently AI application. The study comprised three components, referred to consistently throughout this manuscript as: Study 1, computational readability analysis (assessing technical performance); Study 2, expert clinical review (assessing clinical accuracy and safety); and Study 3, patient preference evaluation (assessing user acceptance and comprehension). The study was registered with the Open Science Framework (OSF; https://osf.io/3cdx6) and is reported with reference to CONSORT-AI guidance where applicable (31). Formal power calculations were not performed; sample sizes (15 expert reviewers and a target of approximately 50 patient participants) were determined pragmatically to balance evaluative breadth against feasibility and are comparable to, or larger than, those of analogous evaluations of AI-generated patient materials (20–22). The adequacy of these sample sizes is considered in the Limitations.

### Intervention

Patiently AI is a mobile application that uses a LLM [GPT-4o, accessed via the OpenAI application programming interface (API)] with task-specific prompt architectures designed for healthcare communication. The application accepts text input or transcribed voice input and generates simplified explanations of clinician-authored medical notes based on user-selected audience (adult, teenager, child, caregiver) and tone (informative, friendly, reassuring). The system supports multiple languages and does not store personal health information. Text submitted to the application is processed via the OpenAI API, under which data are not used for model training. All evaluations in the present study used synthetic notes only, so no real patient data were processed at any stage; regulatory considerations for deployment with identifiable health information are addressed in the Discussion. Each audience–tone configuration applies predefined linguistic strategies, vocabulary constraints, and explanation styles tailored to the intended user group.

### Study 1: computational readability analysis

Ten synthetic medical notes were generated using GPT-4o to simulate common clinical scenarios, including hypertension, diabetes, mental health, oncology, cardiology, neurology, and radiology. Notes were produced from structured prompts specifying the clinical scenario, note type, and characteristic clinical terminology, modeled on common formats of clinical documentation. Each note was reviewed by the author, an experienced medical writer, for clinical plausibility, representative terminology, and complexity consistent with authentic documentation, and was revised where needed before inclusion. The notes were not derived from real patient records or identifiable data; the absence of independent clinician verification of the synthetic notes is acknowledged in the Limitations.

Each synthetic note was processed through Patiently AI using seven audience–tone combinations. Because LLM output is stochastic—the same input and configuration can yield different text on repeated generation—each combination was generated in triplicate to assess the consistency of effects across replicates, yielding a total of 210 AI-generated explanations (10 notes×7 combinations×3 replicates). AI-generated outputs were compared with the original synthetic notes using three established readability metrics. The Flesch–Kincaid Grade Level (FKGL) estimates the United States school grade required to understand a text, based on average sentence length and average syllables per word. Flesch Reading Ease (FRE) scores text from 0 to 100, with higher scores indicating easier reading (60–70 corresponding approximately to plain English). The Gunning Fog Index (GFI) estimates the years of formal education required, weighting sentence length and the density of complex (three-or-more-syllable) words. These metrics were selected because they are among the most widely used and validated measures for patient-facing health materials and capture complementary aspects of surface complexity. The SMOG Index was additionally computed (32), which estimates grade level from polysyllabic word density and is frequently recommended for healthcare materials, was additionally computed programmatically for all texts; because SMOG was developed for passages of 30 or more sentences, values for short texts should be interpreted as approximate. Readability formulas quantify surface-level linguistic complexity (sentence length and word complexity) and do not directly measure semantic or conceptual simplicity; semantic clarity was therefore assessed through expert ratings and patient comprehension questions in Studies 2 and 3, and this distinction is considered in the Limitations. Readability calculations were performed using the Readability Formulas online tool (https://readabilityformulas.com).

### Study 2: expert clinical review

Healthcare professionals were recruited through the author's professional networks and the Prolific research platform (http://www.prolific.com). Eligibility on Prolific was implemented using the platform's occupation-based screening filters for healthcare professionals, together with self-reported professional background and years of experience collected within the survey; independent verification of professional credentials was not performed, and this is acknowledged in the Limitations. Recruitment continued until the target sample of 15 reviewers was reached. Reviewers were independent of the application's development and evaluated AI-generated explanations using an online survey designed by the author for this study, as no existing validated instrument was identified for evaluating audience- and tone-personalized AI outputs; the full instrument is provided as Supplementary Material. All 15 reviewers assessed the same ten AI-generated outputs, purposively selected from the 210 generated explanations to span the four audiences, three tones, and a range of clinical scenarios and severities (10 outputs×15 reviewers = 150 evaluations). Evaluating a common set of outputs enabled assessment of inter-rater agreement.

For each output, presented alongside its source note, reviewers completed one binary clinical safety item (‘Does this output contain any clinically unsafe or misleading information?’; yes indicating a potential safety concern, no indicating clinically safe) and five statements rated on five-point Likert scales anchored at 1 = strongly disagree, 2 = disagree, 3 = neither agree nor disagree, 4 = agree, and 5 = strongly agree: ‘The simplified output preserves the key medical facts’ (medical accuracy); ‘The explanation is clear and understandable’ (clarity); ‘Nothing important is missing’ (completeness); ‘The tone matches the intended audience’ (tone appropriateness); and ‘A patient or carer would trust this explanation’ (trustworthiness). Completeness was thus operationalized as reviewer agreement that no important information from the source note was missing, judged against the original note using professional expertise; no itemized checklist was used. An optional open-ended item (‘Any feedback or concerns about this version?’) followed each output to elicit qualitative feedback on strengths, limitations, and potential risks.

Qualitative responses were analyzed thematically to identify recurrent patterns and concerns; quantitative analysis is described under Statistical analyses below.

### Study 3: patient preference evaluation

Participants were recruited through the Prolific research platform, which provides access to diverse, pre-screened research participants. Inclusion criteria (English fluency and age ≥18 years) were implemented through Prolific's platform-level pre-screening, which draws on participant-registered attributes; no additional independent verification was conducted. Fifty-five participants began the survey and 54 completed it (completion rate 98%). Participants evaluated paired medical notes (original vs. AI-simplified) for five of the ten clinical scenarios: hypertension medication change (adult audience, informative tone), diabetes management (adult, informative), child mental health review (child, friendly), adolescent migraine management (teenager, friendly), and referral for ongoing post-COVID breathlessness (adult, informative). Five scenarios, rather than all ten, were purposively selected to keep survey duration to approximately 15 min and limit respondent fatigue, while spanning different audiences, tones, and levels of clinical seriousness. For each scenario, participants completed four standardized outcome measures, labeled consistently throughout this manuscript and in Table 3: overall preference, rated on a five-point scale (1 = strongly prefer the original note to 5 = strongly prefer the AI-generated explanation), with scores ≥4 classified as preference for the AI-generated explanation; clarity (‘I found this explanation clear and easy to understand’) and confidence in care (‘After reading this, I feel more confident about my care’), each rated from 1 = strongly disagree to 5 = strongly agree; and comprehension, assessed with two or three multiple-choice or true/false questions per scenario about key clinical facts (for example, the newly added medication or the follow-up interval), scored against a predefined answer key. An optional free-text item invited suggestions for further clarification.

The survey concluded with an item assessing likelihood of future use (‘How likely are you to use Patiently AI for simplifying your medical notes?’; 1 = very unlikely to 5 = very likely) and general feedback on the application. The full instrument is provided as Supplementary Material. The evaluation was survey-based and did not involve clinical decision-making or health interventions.

### Statistical analyses

Analyses were performed on the full raw dataset, which is publicly available via the Open Science Framework. Descriptive statistics (means, standard deviations, ranges, and proportions with 95% confidence intervals) summarized all quantitative outcomes at the level of the individual evaluation or response. For readability, original and AI-generated versions were compared using paired *t*-tests at the note level (*n* = 10), with each note's AI-generated value averaged across its 21 outputs to avoid treating replicates as independent observations; Wilcoxon signed-rank tests provided a non-parametric sensitivity analysis, and effect sizes are reported as Cohen's *d*_z_. Consistency across the three generation replicates was assessed by comparing replicate-level means, by Friedman tests across replicates (paired within the 70 note–audience–tone configurations), and by within-configuration standard deviations and two-way mixed-effects consistency intraclass correlation coefficients [ICC(3,1)]. For patient outcomes, one-sample Wilcoxon signed-rank tests compared participant-level mean ratings against the neutral scale midpoint (3); an exact binomial test compared the number of responses preferring the AI-generated version (score ≥4) with those preferring the original (score ≤2); and a Friedman test assessed differences in preference across the five scenarios. Comprehension questions were scored against a predefined answer key, with accuracy reported as the proportion of correct responses. For the expert review, inter-rater agreement across the 15 reviewers rating the common set of ten outputs was quantified using Gwet's AC1 for the binary safety assessment and Gwet's AC2 with linear weights for the Likert-rated domains (33). Gwet's coefficients were selected because they remain interpretable under conditions of high agreement and skewed prevalence, in which kappa statistics and ICCs are known to be paradoxically attenuated (34, 35); ICCs (two-way random-effects, absolute agreement) are additionally reported for completeness. Statistical significance was defined as two-sided *P* < 0.05. Analyses were conducted in Python (SciPy and Pingouin libraries).

### Ethical considerations

This study involved survey-based evaluation of AI-generated explanations using synthetic medical notes and did not include real patient data or clinical interventions. All technical evaluations were conducted using synthetic materials. Expert reviewers and patient participants provided informed consent prior to participation. The study design posed minimal risk to participants and did not involve health-related decision-making.

## Results

### Participant characteristics

The study included 54 patient participants (55 began the survey; completion rate 98%) and 15 expert reviewers. The patient sample was 51.9% male, with the largest age group between 30 and 49 years (40.7%). Most participants reported university-level education (66.7%) and English as the main language spoken at home (94.4%); 74.1% reported living with at least one chronic health condition, and 25.9% identified as caregivers.

The expert panel included medical writers (*n* = 4), general practitioners (*n* = 2), medical specialists (*n* = 2), clinical researchers (*n* = 2), pharmacists (*n* = 1), physiotherapists (*n* = 1), and other healthcare professionals (*n* = 3). Mean professional experience was 8.3 years (range: 1–15 + years).

### Readability outcomes

AI-generated explanations demonstrated consistent improvements in readability across all assessed metrics (Table 1). Mean FKGL decreased from 10.57 to 7.61, corresponding to an improvement of 2.96 grade levels (95% CI: 2.64–3.28). Mean FRE increased from 37.7 to 69.6, an improvement of 31.9 points (95% CI: 29.8–34.0). The GFI decreased from 14.5 to 10.4, representing an improvement of 4.09 points (95% CI: 3.76–4.42), and the SMOG Index decreased from 12.20 to 10.20, an improvement of 2.00 grades (95% CI: 1.69–2.31). All improvements were statistically significant in note-level paired *t*-tests (FKGL: t(9) = 4.74, *P* = 0.001, Cohen's dz = 1.50; FRE: t(9) = 7.44, *P* < 0.001, dz = 2.35; GFI: t(9) = 4.36, *P* = 0.002, dz = 1.38; SMOG: t(9) = 4.22, *P* = 0.002), with Wilcoxon signed-rank sensitivity analyses confirming all comparisons (all *P* ≤ 0.004). Readability outcomes were consistent across the three generation replicates of each configuration: mean FKGL of AI-generated outputs was 7.60, 7.69, and 7.54 across replicates 1–3 (FRE: 69.8, 69.3, 69.7; GFI: 10.43, 10.50, 10.29; SMOG: 10.31, 10.22, 10.07), with no significant differences between replicates (Friedman tests paired within the 70 note–audience–tone configurations; all *P* ≥ 0.061). Between-replicate agreement at the configuration level was moderate to good [ICC(3,1): 0.65–0.85], and mean within-configuration standard deviations across replicates were 0.86 (FKGL), 0.92 (GFI), and 0.68 (SMOG) grade levels and 3.56 FRE points, indicating stable aggregate performance alongside moderate variability between individual generations.

**Table 1:** Comparison of readability metrics between original and AI-generated medical notes.

Metric	Original notes, mean (SD)	AI-generated, mean (SD)	Improvement, mean (95% CI)	*P*-value
FKGL	10.57 (2.84)	7.61 (1.98)	2.96 (2.64–3.28)	0.001
FRE	37.69 (15.42)	69.60 (12.88)	31.90 (29.8–34.0)	<0.001
GFI	14.49 (3.67)	10.40 (2.45)	4.09 (3.76–4.42)	0.002
SMOG	12.20 (1.57)	10.20 (1.82)	2.00 (1.69–2.31)	0.002

Values are presented as mean (SD) unless otherwise stated. Improvements represent differences between original and AI-generated notes, with 95% confidence intervals (CI). *P* values are from note-level paired t-tests (*n* = 10 notes; AI-generated values averaged across the 21 outputs per note). The SMOG Index was computed in response to peer review; SMOG values for short texts are approximate.

SD, standard deviation; CI, confidence interval; FKGL, Flesch–Kincaid Grade Level; FRE, Flesch Reading Ease; GFI, Gunning Fog Index; SMOG, Simple Measure of Gobbledygook.

**Figure 1:**
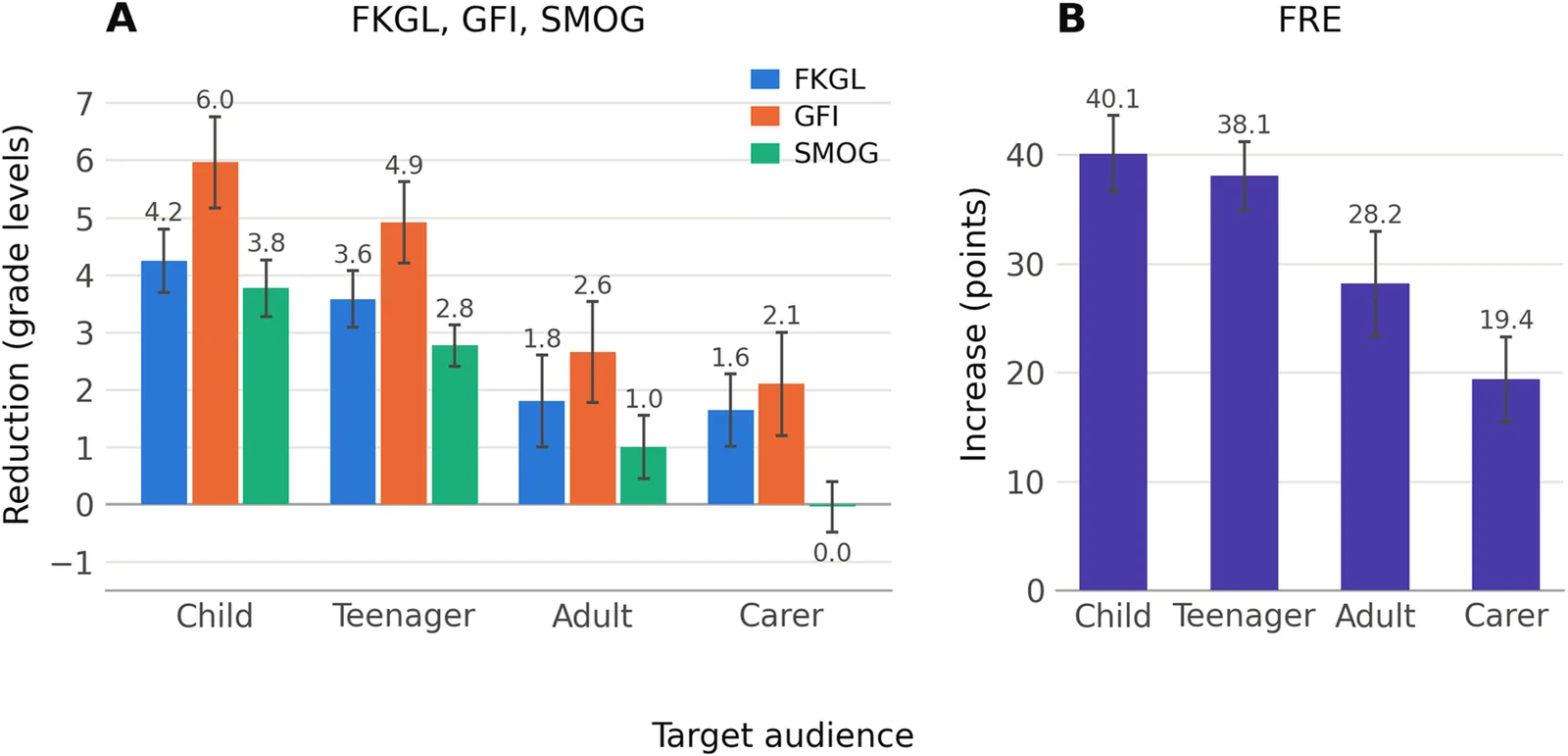
Readability score improvements by target audience. Mean improvements in readability metrics for AI-generated explanations compared with original medical notes, stratified by intended audience and ordered by overall effect size. Panel A shows reductions in the grade-based metrics: Flesch–Kincaid Grade Level (FKGL), Gunning Fog Index (GFI), and SMOG Index. Panel B shows increases in Flesch Reading Ease (FRE; 0–100 scale). Larger reductions in FKGL, GFI, and SMOG and larger increases in FRE indicate greater improvements in readability. Error bars indicate 95% confidence intervals across the AI-generated outputs for each audience.

Readability gains differed by target audience (Figure 1). Child-focused explanations demonstrated the largest reduction in reading level (FKGL reduction: 4.25 grade levels), followed by teenager-focused (3.58), adult-focused (1.80), and carer-focused explanations (1.64). Original medical notes required, on average, a college-level reading ability (FKGL: 10.57). AI-generated explanations achieved an average middle school reading level (FKGL: 7.61), with child-focused explanations reaching elementary school reading levels (6.32). In practical terms, the mean AI-generated output approaches, but does not fully reach, the sixth-grade reading level recommended for patient-facing materials (36); only child-focused explanations approximated this threshold, a gap considered further in the Discussion. SMOG reductions followed the same audience gradient (child: 3.77; teenager: 2.77; adult: 1.00 grades), with essentially no change for carer-focused outputs (−0.04), whose longer, action-oriented explanations retain more polysyllabic vocabulary.

### Expert clinical evaluation

Each of the 15 expert reviewers evaluated the same ten purposively selected outputs, yielding 150 evaluations. Reviewers rated AI-generated explanations positively across all evaluation domains (Table 2). Mean ratings were highest for clarity (4.53 ± 0.77 on a 5-point scale) and medical accuracy (4.49 ± 0.83), followed by completeness (4.39 ± 0.92), tone appropriateness (4.37 ± 0.97), and trustworthiness (4.37 ± 0.90). Inter-rater agreement was substantial for the binary safety assessment (raw agreement 78.1%; Gwet's AC1 = 0.72) and moderate to substantial across the Likert-rated domains (Gwet's AC2 with linear weights: 0.62-0.71), with approximately 90% of individual ratings falling within one point of the item median. ICCs were low across domains, reflecting restricted between-output variance—all ten outputs were rated uniformly favorably—rather than rater inconsistency (34, 35).

**Table 2:** Expert clinical review ratings of AI-generated medical note explanations.

Assessment criterion	Mean score (SD)	Range	High rating (≥4/5)
Medical accuracy	4.49 (0.83)	1.0–5.0	88.7%
Clarity	4.53 (0.77)	1.0–5.0	91.3%
Completeness	4.39 (0.92)	1.0–5.0	84.7%
Tone appropriateness	4.37 (0.97)	1.0–5.0	86.0%
Trustworthiness	4.37 (0.90)	1.0–5.0	86.7%
Clinical Safety	Not applicable	Not applicable	87.3% rated clinically safe

Values are presented as mean (SD) unless otherwise stated. Ratings were provided using five-point Likert scales, where higher scores indicate more favorable assessments. High ratings were defined as scores ≥4. Clinical safety was assessed as a binary outcome and is reported as the proportion of explanations rated as clinically safe. Each of the 15 reviewers rated the same ten outputs; values are computed across all 150 individual evaluations.

SD, standard deviation.

Clinical safety assessments indicated that 87.3% of evaluations (131/150) judged the output to be clinically safe; in the remaining 12.7% (19/150), a potential safety concern was flagged. Identified concerns included excessive simplification that could underemphasize clinical seriousness (*n* = 8), inclusion of general advice not present in the source notes (*n* = 6), and potential for patient misinterpretation (*n* = 5). Safety flags were distributed unevenly across outputs: nine of the ten reviewed outputs received at least one flag, with the largest numbers for a carer-focused, reassuring-tone explanation of falls related to orthostatic hypotension (5/15 reviewers), a teenager-focused informative explanation of colorectal cancer surveillance (3/15), and an adult-focused informative diabetes explanation (3/15); the adult-focused hypertension explanation received none. Outputs written for child audiences received few flags overall (three across two outputs), although reviewer comments cautioned that strongly simplified, metaphor-based framing of serious diagnoses for young audiences risks underemphasizing clinical seriousness.

Qualitative feedback highlighted clarity and accessibility as common strengths. Reviewers noted that explanations were “*well written, clear and understandable by most people*” and that outputs generally preserved key medical facts while aligning with the intended audience and tone. Some reviewers suggested that reassurance-oriented explanations could be shortened to improve readability in certain contexts.

### Patient preference and acceptance

Patient evaluation demonstrated a consistent preference for AI-generated explanations (Table 3). The mean preference score across the 270 scenario responses was 3.89 (SD 1.32), significantly above the neutral midpoint (one-sample Wilcoxon signed-rank test on participant means, *P* < 0.001). Of the 270 responses, 189 (70.0%) indicated preference for the AI-generated explanation (score ≥4), vs. 56 (20.7%) preferring the original (score ≤2; exact binomial test, *P* < 0.001). Preference differed significantly across scenarios [Friedman *χ*^2^(4) = 42.65, *P* < 0.001], ranging from 3.41 (chronic migraine, teenager audience) and 3.46 (mental health, child audience) to 4.30 (post-COVID symptoms, adult audience).

**Table 3:** Patient preference and satisfaction results for AI-generated medical note explanations.

Outcome measure	Mean score (SD)	Range	Positive response (≥4/5)
Overall Preference	3.89 (1.32)	1.0–5.0	70.0%
Clarity	4.58 (0.65)	1.0–5.0	93.7%
Confidence in Care	4.19 (0.85)	1.0–5.0	77.0%
Likelihood to Use	3.83 (1.24)	1.0–5.0	70.4%
Comprehension (correct responses)	98.1% (583/594)	94.4%–100% by scenario	Not applicable

SD, standard deviation.

Values are presented as mean (SD) unless otherwise stated. All ratings use five-point Likert scales, where higher scores indicate more favorable responses. Comprehension is reported as the percentage of correct responses to scenario-specific factual questions (54 participants×11 questions); all other measures summarize the 270 scenario responses (54 participants×5 scenarios), except likelihood to use, asked once per participant (*n* = 54).

Participants rated AI-generated explanations highly for clarity (mean: 4.58 ± 0.65), indicating that the explanations were easy to understand. Confidence in care was also rated positively (mean: 4.19 ± 0.85), suggesting that simplified explanations did not reduce perceived trust in medical care. Both ratings were significantly above the scale midpoint (*P* < 0.001).

Comprehension assessments showed high accuracy across scenarios, with 98.1% of responses correct overall (583/594; scenario range 94.4%–100%). Participants accurately identified key clinical information, including medications, follow-up timelines, and care instructions.

Reported usage intentions were favorable. Overall, 70.4% of participants indicated a likelihood of using Patiently AI to simplify their medical notes (score ≥4), with a mean likelihood score of 3.83 (SD 1.24), significantly above the scale midpoint (*P* < 0.001).

### Audience-specific performance

Analysis by target audience revealed systematic differences in readability outcomes and user responses. Child-focused explanations achieved the largest readability improvements but were more frequently associated with expert concerns regarding appropriateness for serious medical conditions. Teenager-focused explanations demonstrated a balance between reduced complexity and clinical seriousness. Adult-focused explanations maintained professional tone while improving accessibility, and carer-focused explanations emphasized actionable information and support.

**Figure 2:**
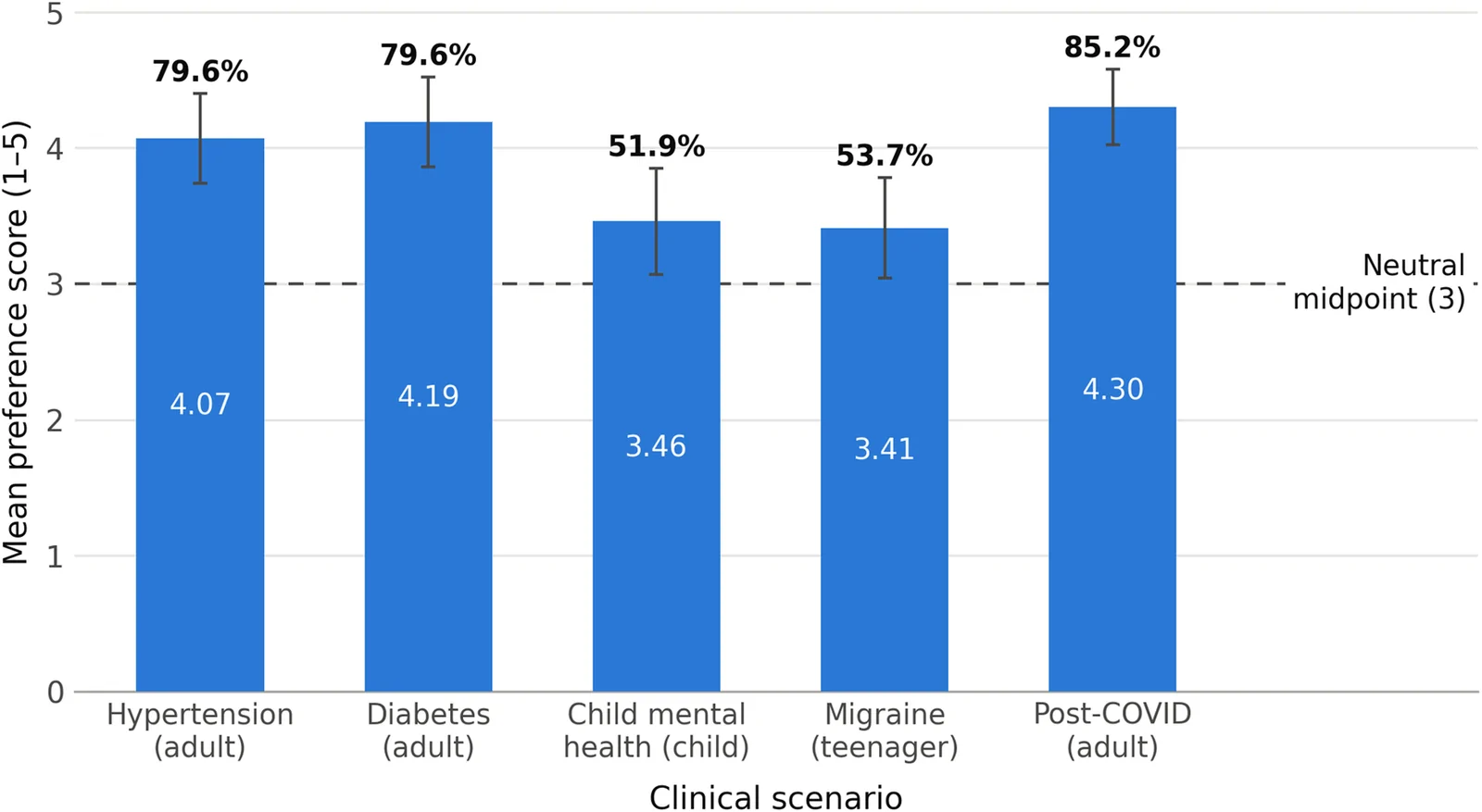
Patient preference for AI-generated explanations by clinical scenario. Mean preference scores for AI-generated explanations across five clinical scenarios, rated on a five-point Likert scale (1 = strong preference for original notes; 5 = strong preference for AI-generated explanations). Percentages above bars indicate the proportion of participants expressing a positive preference for AI-generated explanations (score ≥4). Error bars indicate 95% confidence intervals, and the dashed horizontal line marks the neutral midpoint of the preference scale (3). Scenarios were: (1) hypertension management (ramipril added for persistently elevated blood pressure); (2) diabetes management (consideration of GLP-1 therapy for elevated HbA1c); (3) child mental health review (sertraline initiated for mood and sleep disturbance, no suicidal ideation); (4) chronic migraine management (topiramate initiation and headache diary); and (5) referral for ongoing post-COVID breathlessness.

Patient preferences varied by scenario and audience. Adult-focused explanations received consistently high ratings across clinical contexts. Child- and teenager-focused explanations were rated favorably for age-appropriate scenarios but less preferred in situations involving serious or complex conditions. As illustrated in Figure 2, more than 70% of participants preferred AI-generated explanations in three of five scenarios, with mean preference ratings above 4.0 in three scenarios. The highest preference was observed for scenario 5 (mean score: 4.30; 85.2% preferring AI-generated explanations).

## Discussion

This study evaluated a deliberately constrained, language-focused AI application designed to personalize explanations of clinician-authored medical notes. Across technical, expert, and patient-centered measures, the findings indicate that AI-generated explanations can improve accessibility of medical documentation while preserving clinical content and safety when applied within clearly defined boundaries.

Substantial improvements in readability were observed across all evaluated metrics. Reductions of nearly three grade levels shifted medical notes from college-level complexity to approximately middle school readability, a range commonly recommended for patient-facing health materials (37). Nevertheless, the mean AI-generated output (FKGL 7.61) remains above the sixth-grade level recommended by the American Medical Association for patient materials (36), and only child-focused outputs (mean FKGL 6.32) approximated that threshold. For readers with limited literacy, text at a seventh-to-eighth-grade level may still present a barrier, suggesting that the strongest simplification settings—currently framed for younger audiences—could also serve adults with low literacy if reframed with age-appropriate content and tone. These gains were most pronounced in child- and teenager-focused explanations, reflecting systematic adaptation of linguistic complexity based on intended audience. Importantly, improvements extended beyond simple sentence shortening and reflected adjustments in vocabulary, framing, and explanatory structure, suggesting that audience-specific tailoring can be achieved without loss of core medical information.

Expert clinical review demonstrated generally high ratings for medical accuracy, clarity, and trustworthiness. A total of 87.3% of AI-generated explanations were rated as clinically safe by expert reviewers. While this compares favorably with prior evaluations of AI-generated patient materials (22), the remaining 12.7% of outputs flagged for potential safety concerns warrant careful consideration. These concerns primarily reflected over-simplification of serious conditions or the inclusion of generalized advice not present in the source notes, rather than factual inaccuracies or hallucinated clinical content. This pattern suggests that residual risks relate to boundary management and communication framing, reinforcing the importance of constrained system design and appropriate clinical oversight. The distribution of safety flags suggests concrete risk-management practices for clinicians and educators: outputs should be verified against the source note before being shared—a human verification step made necessary by the inherent stochasticity of LLM generation, which motivated the triplicate design and means that individual outputs cannot be assumed uniformly safe even when average performance is high. Particular caution should apply to reassuring-tone, carer-directed explanations of prognostically uncertain findings and to child-audience framings of serious diagnoses, the configurations that attracted the most safety concerns. AI-generated content should be explicitly labeled as such, and users should retain a clear route back to the clinical team for questions.

The preservation of clinical accuracy alongside improved accessibility addresses a common concern that simplification may compromise informational integrity. Expert ratings for medical accuracy and completeness indicate that AI-generated explanations were largely successful in retaining essential content while presenting it in a more understandable form. This aligns with emerging evidence that AI-assisted communication tools may support, rather than undermine, high-quality patient education when appropriately constrained (38). Beyond readability and accuracy, the potential benefits of validated AI communication tools warrant consideration within the broader literature. Automated generation of lay-language explanations could reduce the time clinicians spend drafting patient-facing summaries, a benefit reported in evaluations of AI-drafted test-result explanations (38) and discharge-summary transformation (20). Integrated into patient portals or shared alongside released notes, such tools could support comprehension and recall at the moment patients access their records, complementing rather than disrupting clinical workflow. By adapting language to the reader's needs and role, they may also foster more patient-centered communication, which is itself associated with improved engagement and outcomes (28). Realizing these benefits at scale, however, depends on the safeguards described here.

Patient evaluations demonstrated consistent preference for AI-generated explanations across scenarios, with high ratings for clarity and comprehension. Notably, improved understanding was associated with increased confidence in care, suggesting that clearer explanations may enhance patient trust rather than generate confusion or doubt. Reported willingness to use the application indicates acceptance of AI-supported communication tools, although real-world adoption and sustained engagement remain to be evaluated. Preference nevertheless differed markedly across scenarios. The two scenarios with the lowest preference—child mental health (3.46) and chronic migraine (3.41)—were precisely those presented in child- and teenager-focused framings, whereas the three adult-framed scenarios scored between 4.07 and 4.30. The most plausible explanation is audience mismatch: adult survey participants evaluated explanations written for younger readers, and strongly simplified, informal framing may be perceived as less appropriate, or even infantilizing, when clinical content is serious. Clinical seriousness itself may also reduce acceptance of simplification, consistent with expert concerns about child-focused framings of serious diagnoses. Evaluation with age-matched audiences (for example, adolescents rating teenager-focused outputs, or parents rating child-focused outputs intended for their children) is an important direction for future work.

Audience-specific analyses highlighted important nuances. While child-focused explanations achieved the largest readability gains, expert feedback indicated greater caution may be required when communicating serious diagnoses to younger audiences. Teenager- and adult-focused explanations were more consistently rated as appropriate across scenarios, underscoring the importance of contextual judgment when applying audience adaptations. These findings support the view that AI communication tools should complement, rather than replace, clinician judgment, particularly for vulnerable populations. At the same time, the audience-adaptation architecture evaluated here offers a foundation for personalized communication for patients with limited literacy or cognitive impairment: the same parameterization that generates child-appropriate language could be extended with configurations validated for easy-read and cognitively accessible formats, developed through co-design with the relevant populations and deployed with the human verification safeguards described above.

Several limitations should be acknowledged. No formal power calculation informed the sample sizes, which were determined pragmatically. The expert panel, although multidisciplinary, was relatively small and may not reflect the full range of clinical perspectives; expert eligibility relied on Prolific's occupation-based screening and self-reported background without independent credential verification, and completeness judgments were made without an itemized checklist. The synthetic notes were reviewed for plausibility by the author but not independently verified by practicing clinicians. Readability formulas capture surface-level linguistic complexity rather than semantic or conceptual simplicity; semantic clarity was assessed only indirectly, through expert clarity and completeness ratings and a small number of comprehension questions per scenario, and was not evaluated separately across patient literacy levels. Patient evaluation was conducted in a survey-based setting using simulated scenarios rather than embedded within real clinical workflows. Actual use patterns, comprehension outcomes, and downstream effects on care decisions may differ in practice. In addition, the study relied on synthetic medical notes to ensure privacy and controlled evaluation. While this approach enabled systematic testing, further validation using authentic clinical documentation is warranted to confirm generalizability. Finally, evaluations focused on English-language outputs in a predominantly university-educated participant sample, limiting conclusions regarding other languages, cultural contexts, or populations with very low health literacy. Participants were recruited through an online research platform and may be more digitally literate or comfortable with technology than the general patient population (39). Although the application supports multiple languages, the present evaluation focused on English-language outputs, and performance across other linguistic and cultural contexts remains to be established. Finally, the study assessed understanding and preferences in a survey-based setting rather than during live clinical encounters, and real-world use may introduce additional contextual factors not captured here.

The broader ethical context of AI-mediated patient communication also merits consideration. Recent reviews have highlighted risks related to transparency, over-anthropomorphization of AI systems, and inappropriate reliance on AI-generated information (23). The findings of the present study reinforce the importance of clear scope definition, explicit labeling of AI-generated content, and appropriate clinical governance. Communication-focused AI tools should be distinguished from diagnostic or decision-support systems, with oversight frameworks proportionate to their intended function.

From a policy and implementation perspective, validated AI communication tools may offer a scalable complement to existing health literacy interventions. Integration into patient portals or educational workflows, accompanied by clinician oversight and patient education about system limitations, may support broader access to understandable medical information while maintaining safety. Deployment also raises data-protection obligations. Although the present evaluation processed only synthetic notes, real-world use involves submitting user-entered clinical text to a third-party API. Patiently AI accesses GPT-4o via the OpenAI API, under which submitted data are not used for model training, and the application does not store personal health information; nonetheless, integration with identifiable health records in regulated settings would require jurisdiction-specific safeguards—for example, a business associate agreement under HIPAA in the United States, or equivalent arrangements under the UK GDPR—as a precondition of clinical deployment.

## Conclusions

This mixed-methods validation study demonstrates that a deliberately constrained AI application can improve the accessibility of medical notes while preserving clinical accuracy and safety. Patiently AI achieved meaningful reductions in reading complexity, favorable expert evaluations, and high patient acceptance across diverse scenarios and audiences. These findings support the potential role of AI-powered communication tools in addressing persistent health literacy challenges when applied within clearly defined, non-interpretive boundaries. Future work should focus on real-world implementation, longitudinal outcomes, and evaluation across broader linguistic and cultural contexts to inform responsible deployment in clinical practice.

## Data Availability

The datasets presented in this study can be found in online repositories. The names of the repository/repositories and accession number(s) can be found below: Repository: Open Science Framework (OSF) Direct link: https://osf.io/3cdx6 Accession/identifier: OSF DOI: 10.17605/OSF.IO/3CDX6.
