# Supplementary material for: Validation of an AI-powered mobile application for personalizing medical note explanations: a mixed-methods evaluation
Source: Front Digit Health. 2026 Aug 25;8:1771051. doi: 10.3389/fdgth.2026.1771051 (PMC13550179; doi:10.3389/fdgth.2026.1771051)
Supplement: Supplementary file 1 [file Datasheet1.docx]

Supplementary Material

Validation of an AI-powered mobile application for personalizing medical note explanations: a mixed-methods evaluation (Manuscript ID 1771051)

This supplement reproduces the two survey instruments used in the study. Both were administered online. Raw response data are available via the Open Science Framework (https://doi.org/10.17605/OSF.IO/3CDX6).

# Supplementary Material S1. Expert clinical review survey

Administered to healthcare professionals (n = 15). After demographic items, each reviewer was shown the same ten AI-generated outputs (each presented alongside its source note) and completed the identical question battery for each output.

## Demographic items

- Name (optional)
- Prolific ID
- Professional background
- Years of practice / experience (optional)

## Question battery (repeated for each of the ten outputs)

- 1. Does this output contain any clinically unsafe or misleading information? (Yes / No)
- 2–6. Five-point Likert items (1 = strongly disagree, 2 = disagree, 3 = neither agree nor disagree, 4 = agree, 5 = strongly agree):
- “The Simplified Output preserves the key medical facts” *(medical accuracy)*
- “The explanation is clear and understandable” *(clarity)*
- “Nothing important is missing” *(completeness)*
- “The tone matches the intended audience” *(tone appropriateness)*
- “A patient or carer would trust this explanation” *(trustworthiness)*
- 7. Any feedback or concerns about this version? (open text, optional)

## The ten evaluated outputs

### Output 1: (Audience: Adult; Tone: Informative)

Your blood pressure is not as well-managed as we would like, even though you're taking amlodipine at a dose of 10 mg each day. To help improve your blood pressure control, we are going to add another medication called ramipril at a dose of 2.5 mg. We'll check back in with you in 6 weeks to see how things are going.

In the meantime, please keep up with any lifestyle changes we've discussed, such as eating a balanced diet, exercising regularly, and reducing salt intake. Also, continue to monitor your blood pressure at home. This will help us see how your blood pressure is doing day-to-day and make any necessary adjustments to your treatment plan.

### Output 2: (Audience: Carer; Tone: Reassuring)

As a family caregiver, it's important to understand that your loved one has a small, 1.2 cm area in their liver that was discovered during a CT scan. This area is less dense than the surrounding tissue, which could mean it’s a harmless cyst or a type of blood vessel growth called a hemangioma.

Here’s what you can do:

1. Stay Calm and Informed: Right now, there's no need for any immediate medical treatment, which is good news. This means the doctors believe it's not an urgent issue.

2. Monitor and Note: Keep track of any changes in your loved one’s health, such as new symptoms like pain in the abdomen, unexplained weight loss, or a noticeable change in their overall well-being.

3. Plan for Follow-Up: The doctors recommend another scan in 3 months to see if there are any changes. Make sure to mark this on your calendar and plan any necessary appointments.

4. Communicate: Keep open lines of communication with your loved one and their healthcare team. If you notice any new or worsening symptoms, or if you have questions, don’t hesitate to reach out to the doctor.

5. Self-Care for You: Caring for someone else is important, but so is taking care of yourself. Make time for activities that help you relax and recharge, whether it’s a short walk, reading a book, or spending time with friends.

6. Seek Support: If you feel overwhelmed, consider joining a support group for caregivers. Sharing experiences and advice with others in a similar situation can be incredibly comforting.

Remember, by staying organized and informed, you’re doing a great job in supporting your loved one’s health journey. If anything seems unusual or you're unsure about what to do, don't hesitate to seek professional advice. You’ve got this!

### Output 3: (Audience: Teenager; Tone: Informative)

Hey there! So, Mr. X had a surgery in 2021 to remove part of his colon, which is called a right hemicolectomy. Now, he's getting regular check-ups to make sure everything's okay with his colon health. Recently, his CEA levels, which are markers doctors check to see how things are going, are normal. That means there's nothing new or worrying happening with his health right now. His next big check, called a colonoscopy, is planned for 2026. This kind of regular monitoring is like checking on your car to make sure it runs smoothly. It's important for keeping everything in good shape. Staying on top of his health like this is setting him up for a healthier future!

### Output 4: (Audience: Adult; Tone: Informative)

Your HbA1c level, which is a measure of your average blood sugar over the past two to three months, is still high at 72 mmol/mol. This suggests that your blood sugar levels have been consistently above the normal range. We've talked before about the importance of sticking to your recommended diet and getting regular exercise, and we encourage you to continue focusing on these areas.

If we don't see an improvement in your HbA1c levels over the next three months, we may need to consider starting a new type of medication called GLP-1 therapy. This medication helps lower blood sugar levels and can also aid in weight loss. We'll discuss this option further if necessary, but for now, let's concentrate on your diet and exercise routine to help bring your blood sugar levels down.

### Output 5: (Audience: Carer; Tone: Informative)

As a family caregiver, it's important to understand the current health status and care plan for your loved one. The echocardiogram results indicate that the heart's left ventricle, which is responsible for pumping blood, is functioning well. There is a mild thickening of the heart muscle, known as left ventricular hypertrophy (LVH), but nothing concerning with the heart valves.

Here's what you need to do:

1. Medication Management: Continue administering bisoprolol as prescribed. This medication helps manage heart conditions by slowing the heart rate and reducing blood pressure.

2. Monitor for Symptoms: Keep an eye out for any new or worsening symptoms such as shortness of breath, chest pain, fatigue, dizziness, or swelling in the legs and ankles. These could indicate changes in the heart condition that need medical attention.

3. Regular Check-ins: Maintain regular appointments with the healthcare provider to monitor heart health and adjust the care plan if necessary.

4. Lifestyle Support: Encourage a heart-healthy lifestyle that includes a balanced diet, regular physical activity, stress management, and smoking cessation if applicable.

5. When to Seek Help: If you notice any of the symptoms mentioned above or if your loved one experiences an unexpected change in their health, contact a healthcare professional immediately.

6. Self-Care for the Caregiver: It's essential to take care of yourself as well. Ensure you get enough rest, eat well, find time for activities you enjoy, and seek support from friends, family, or caregiver support groups.

Remember, you're doing an important job, and your attentiveness plays a crucial role in the health and well-being of your loved one.

### Output 6: (Audience: Child; Tone: Friendly)

Emily is feeling happier, but she is still having trouble sleeping well. She told us she’s not having any thoughts about hurting herself, which is good. We are starting her on a small dose of medicine called sertraline, like giving her a little helper to feel better. She will take it every day. We will meet with her again in 4 weeks to see how she’s doing, just like checking in with a friend to make sure everything is okay.

### Output 7: (Audience: Carer; Tone: Reassuring)

As a caregiver for Mrs. X, it's important to be aware that her recent falls might be due to a condition called orthostatic hypotension, which means her blood pressure drops when she stands up. This can be made worse by some of her medications, like amitriptyline, furosemide, and perindopril. We are currently reviewing her medication plan to address this.

In the meantime, here are some practical steps you can take to help prevent falls and ensure her safety:

1. Stand Up Slowly: Encourage Mrs. X to rise slowly from a sitting or lying position. This gives her body time to adjust and can help prevent dizziness.

2. Stay Hydrated: Ensure she drinks plenty of fluids unless advised otherwise by her doctor, as staying well-hydrated can help maintain blood pressure levels.

3. Monitor Her Medications: Keep a list of her medications and note any changes in her condition. If you notice increased dizziness or more frequent falls, contact her healthcare provider.

4. Home Safety: Keep the home environment safe by removing tripping hazards like loose rugs or clutter. Ensure good lighting in all areas, especially near stairs.

5. Occupational Therapy: An occupational therapist will be visiting to assess her needs and may recommend aids or modifications to help her move around safely.

Signs to Watch For:

- Sudden dizziness or lightheadedness

- Frequent falls or balance issues

- Noticeable changes in her alertness or confusion

When to Seek Professional Help:

- If she experiences a fall and is injured

- If dizziness is severe or does not improve

- If there are any changes in her ability to perform daily activities

Self-Care for You, the Caregiver:

Taking care of yourself is just as important. Make sure to:

- Take breaks when needed and ask for help from family or friends.

- Maintain your own health with regular check-ups and a balanced diet.

- Reach out to support groups or counseling if you feel overwhelmed.

Your dedication and care for Mrs. X are invaluable, and you're doing a wonderful job. Remember, help is always available, and you're not alone in this journey.

### Output 8: (Audience: Teenager; Tone: Friendly)

Hey there! It looks like the type of headaches you're experiencing match what we call chronic migraines. To help manage these, we're going to start a treatment with a medication called topiramate. Think of it like a shield that helps reduce how often you get these headaches.

Also, it would be super helpful if you could keep a little journal or diary of when you get headaches. It’s like a detective tool to help us figure out what might be causing them. Plus, try to steer clear of things you know trigger these headaches for you—like certain foods, not getting enough sleep, or too much screen time. This way, we can work together to make you feel better and keep those headaches at bay!

### Output 9: (Audience: Child; Tone: Reassuring)

Hey there! So, imagine your body is like a superhero team, and each part has a special job. Sometimes, we need to check on these parts to make sure they're doing okay.

Right now, we're talking about a part called the prostate, which is like a little helper inside the body. We did a tiny test called a biopsy to see how it's doing. Think of it as taking a small peek to make sure everything is alright. We found something called adenocarcinoma, which is a kind of bump that needs some attention.

Next, we're going to use some cool picture-taking machines, like a CT scan, to take a closer look inside and see what's happening. It's like taking superhero x-ray vision to make sure everything's in its right place.

After that, a team of super smart doctors will come together to chat about the best way to help. It’s like having a meeting of superheroes to decide the best plan to keep the body strong.

Remember, we have a plan and a team to take care of everything, and we'll keep you and your family updated on what comes next. You’re not alone, and there’s nothing to worry about!

### Output 10: (Audience: Adult; Tone: Informative)

You’ve mentioned that you’re experiencing ongoing breathlessness, especially when you exert yourself, like when you walk or climb stairs. This is likely due to a virus you had before. The good news is that we didn’t find any signs of a blood clot in your lungs in your recent CT scan. To help you manage these symptoms, we’re going to refer you to a clinic that specializes in long COVID recovery. They’ll be able to provide more support and guidance to improve your breathing and overall well-being.

# Supplementary Material S2. Patient preference evaluation survey

Administered to adult participants recruited via Prolific (n = 54 completers). After demographic items, participants viewed five scenario blocks. In each block the original clinical note and the AI-generated explanation were shown side by side, followed by the questions listed.

## Demographic items

- Gender
- What is your age?
- Highest level of education completed
- Main language spoken at home
- Do you have any chronic health conditions?
- Are you a caregiver for someone with health conditions?

## Per-scenario question battery

- Which version did you prefer? (1 = strongly prefer the original note, 3 = no preference, 5 = strongly prefer the AI-generated explanation)
- Brief reason for your preference (open text)
- “I found this explanation clear and easy to understand” (1 = strongly disagree to 5 = strongly agree)
- “After reading this, I feel more confident about my care” (1 = strongly disagree to 5 = strongly agree)
- What, if anything, would you like clarified or explained further? (open text, optional)
- Two or three scenario-specific comprehension questions (multiple choice / true-false; listed with each scenario below)

### Scenario 1: Hypertension medication change — (Audience: Adult; Tone: Informative)

**Original note:**

Blood pressure remains suboptimally controlled despite amlodipine 10 mg daily. We will add ramipril 2.5 mg and reassess in 6 weeks. Advised to continue lifestyle modifications and home BP monitoring.

**AI-generated explanation:**

Your blood pressure is not as well-managed as we would like, even though you're taking amlodipine at a dose of 10 mg each day. To help improve your blood pressure control, we are going to add another medication called ramipril at a dose of 2.5 mg. We'll check back in with you in 6 weeks to see how things are going.

In the meantime, please keep up with any lifestyle changes we've discussed, such as eating a balanced diet, exercising regularly, and reducing salt intake. Also, continue to monitor your blood pressure at home. This will help us see how your blood pressure is doing day-to-day and make any necessary adjustments to your treatment plan.

**Comprehension questions:**

- Based on the information provided, what new medication is being added to your treatment? (multiple choice)
- Based on the information provided, when is your next appointment? (multiple choice)
- True or False: You should stop monitoring your blood pressure at home

### Scenario 2: Diabetes management (HbA1c) — (Audience: Adult; Tone: Informative)

**Original note:**

HbA1c remains elevated at 72 mmol/mol despite previous advice. The importance of dietary adherence and regular exercise was reinforced. We are considering escalation to GLP-1 therapy if there is no improvement over the next 3 months

**AI-generated explanation:**

Your HbA1c level, which is a measure of your average blood sugar over the past two to three months, is still high at 72 mmol/mol. This suggests that your blood sugar levels have been consistently above the normal range. We've talked before about the importance of sticking to your recommended diet and getting regular exercise, and we encourage you to continue focusing on these areas.

If we don't see an improvement in your HbA1c levels over the next three months, we may need to consider starting a new type of medication called GLP-1 therapy. This medication helps lower blood sugar levels and can also aid in weight loss. We'll discuss this option further if necessary, but for now, let's concentrate on your diet and exercise routine to help bring your blood sugar levels down.

**Comprehension questions:**

- Based on the information provided, what does HbA1c measure? (multiple choice)
- Based on the information provided, what medication might be considered if no improvement? (multiple choice)

### Scenario 3: Child mental health review — (Audience: Child; Tone: Friendly)

**Original note:**

Emily reports an improved mood but continues to experience disrupted sleep. She denies any suicidal ideation. We are initiating sertraline at 25 mg daily. A follow-up with CAMHS is scheduled in 4 weeks

**AI-generated explanation:**

Emily is feeling happier, but she is still having trouble sleeping well. She told us she’s not having any thoughts about hurting herself, which is good. We are starting her on a small dose of medicine called sertraline, like giving her a little helper to feel better. She will take it every day. We will meet with her again in 4 weeks to see how she’s doing, just like checking in with a friend to make sure everything is okay.

**Comprehension questions:**

- Based on the information provided, what medication is Emily starting? (multiple choice)
- Based on the information provided, when is the follow-up appointment? (multiple choice)

### Scenario 4: Adolescent chronic migraine — (Audience: Teenager; Tone: Friendly)

**Original note:**

Headache pattern is consistent with a diagnosis of chronic migraine. We will initiate prophylactic treatment with topiramate. The patient has been advised to keep a headache diary and avoid known triggers

**AI-generated explanation:**

Hey there! It looks like the type of headaches you're experiencing match what we call chronic migraines. To help manage these, we're going to start a treatment with a medication called topiramate. Think of it like a shield that helps reduce how often you get these headaches.

Also, it would be super helpful if you could keep a little journal or diary of when you get headaches. It’s like a detective tool to help us figure out what might be causing them. Plus, try to steer clear of things you know trigger these headaches for you—like certain foods, not getting enough sleep, or too much screen time. This way, we can work together to make you feel better and keep those headaches at bay!

**Comprehension questions:**

- Based on the information provided: what type of treatment is topiramate? (multiple choice)
- Based on the information provided, what should you keep track of? (multiple choice)

### Scenario 5: Post-COVID breathlessness referral — (Audience: Adult; Tone: Informative)

**Original note:**

The patient reports ongoing exertional breathlessness, which is likely post-viral in origin. There is no evidence of pulmonary embolism on recent CTPA. We will proceed with a referral to the long COVID clinic

**AI-generated explanation:**

You’ve mentioned that you’re experiencing ongoing breathlessness, especially when you exert yourself, like when you walk or climb stairs. This is likely due to a virus you had before. The good news is that we didn’t find any signs of a blood clot in your lungs in your recent CT scan. To help you manage these symptoms, we’re going to refer you to a clinic that specializes in long COVID recovery. They’ll be able to provide more support and guidance to improve your breathing and overall well-being.

**Comprehension questions:**

- Based on the information provided, what test ruled out blood clots? (multiple choice)
- Based on the information provided, where are you being referred? (multiple choice)

## Closing item

- Finally, how likely are you to use Patiently AI for simplifying your medical notes? (1 = very unlikely to 5 = very likely)
